# The silent burden: Alexithymia and quality of life in acromegaly patients

**DOI:** 10.1007/s12020-026-04606-7

**Published:** 2026-04-02

**Authors:** Lala Soltanova, Ahmet Bera Aygun, Banu Betul Kocaman, Ilkin Muradov, Sabriye Sibel Taze, Senol Turan, Pinar Kadioglu, Emre Durcan

**Affiliations:** 1 Division of Endocrinology and Metabolic Diseases, Department of Internal Medicine, Cerrahpasa Faculty of Medicine, Istanbul University-Cerrahpasa, Istanbul, Türkiye Turkey; 2 Department of Internal Medicine, Cerrahpasa Faculty of Medicine, Istanbul University- Cerrahpasa, Istanbul, Türkiye Turkey; 3 Department of Psychiatry, Cerrahpasa Faculty of Medicine, Istanbul University- Cerrahpasa, Istanbul, Türkiye Turkey; 4 Division of Endocrinology, Metabolism and Diabetes, Department of Internal Medicine Nursing Service, Cerrahpasa Faculty of Medicine, Istanbul University-Cerrahpasa, Istanbul, Türkiye Turkey; 5 Pituitary Center, Istanbul University-Cerrahpasa, Istanbul, Türkiye Turkey

**Keywords:** Acromegaly, alexithymia, body image, depression, anxiety

## Abstract

**Background:**

We aim to investigate the potential link between the physical changes associated with acromegaly and the development of alexithymia, as well as its related psychopathological outcomes and impact on quality of life.

**Methods:**

The study included 137 patient with acromegaly and 125 control group patients. Body Image Scale (BIS), Toronto Alexithymia Score-20 (TAS-20), Hospital Anxiety and Depression inventory (HAD), Short Form-36 (SF-36) and Acromegaly Quality of Life Scale (AcroQol) were used to the patients.

**Results:**

In the acromegaly group, TAS-20 total scores, as well as the Difficulty Identifying Feelings (DIF) and Difficulty Describing Feelings (DDF) subscale scores, were higher compared to the control group (51 vs. 44, *p* < 0.01; 16 vs. 12, *p* < 0.01; and 12 vs. 11, *p* < 0.01, respectively). In contrast, no notable difference was observed in the Externally Oriented Thinking (EOT) scores between the groups. The prevalence of alexithymia was markedly higher in individuals with acromegaly (16.7%) than in the control group (3.2%, *p* < 0.001). A positive correlation was found between alexithymia and both depression and anxiety scores (*r* = 0.469, *p* < 0.001; *r* = 0.377, *p* < 0.01, respectively). In contrast, alexithymia was negatively correlated with SF-36, AcroQoL and BIS (*r* = − 0.426, *p* < 0.01; *r* = − 0.465, *p* < 0.01; *r* = − 0.364, *p* < 0.01, respectively). Patients with acromegaly reported poorer body image and a higher rate of impaired body perception compared to control group (43.7% vs. 19.2%, *p* < 0.01).

**Conclusion:**

The present study demonstrates that alexithymia is more prevalent in patients with acromegaly compared to controls and often persists even after remission.

## Introduction

Acromegaly is a long-standing disease caused by endocrine dysfunction and associated with multisystem involvement, including the cardiovascular, respiratory, and neurological systems [1, 2]. The management of acromegaly involves not only achieving biochemical remission and addressing physiological complications, but also improving patients’ quality of life through a multidisciplinary approach that includes psychological care [3–5]. Psychosocial factors such as depression, anxiety, body image, disease perception, and sleep quality affect the quality of life of these patients. Most of these factors are modifiable and can provide valuable targets for future interventions [6].

Psychiatric comorbidities, particularly depression and anxiety, are highly prevalent among patients with acromegaly, affecting approximately 40–50% of cases [7, 8]. Despite their high prevalence and substantial impact on patients’ daily functioning, these conditions often remain underdiagnosed [6]. Persistent hormonal imbalances, physical deformities, arthropathies, and the chronic, progressive nature of the disease often contribute to the exacerbation of depression and anxiety [9].

Body image is another important factor closely related to both physical and mental health, notably influencing individuals’ emotions, thoughts, behaviors [10]. It is defined as a person’s perception of their own body and is particularly shaped by their awareness of others’ reactions to their appearance, body shape, and bodily functions [11]. In patients with acromegaly, the physical changes caused by the disease such as facial and bodily alterations are thought to negatively affect body image [12]. This altered perception can contribute to reduced self-confidence, emotional suppression, and social isolation, further compromising psychological well-being [13].

Alexithymia described as emotional “blindness”, a personality structure characterized by difficulty understanding one’s own emotions, distinguishing them from bodily arousal signals, and verbally expressing their emotions to others [14, 15]. Individuals with alexithymia are known to have low levels of empathy, exhibit impulsive behavior, and experience difficulties in establishing and maintaining meaningful interpersonal relationships [16, 17]. These interpersonal difficulties may stem from limited emotional awareness, reduced capacity for emotional expression, the frequent use of negative emotion words in describing relationships, and difficulties in social and emotional interpretation [18]. In patients with acromegaly, physical changes caused by the disease, can lead to loss of self-confidence, social isolation and emotional suppression [19, 20]. Alexithymia, which is a transdiagnostic risk factor for depression, anxiety and post-traumatic stress disorder, may be a psychological risk factor for the quality of life of patients with acromegaly [8, 21].

AcroQol: Acromegaly Quality of life Questionnaire.

Recent systematic reviews have shown that evidence regarding alexithymia in patients with acromegaly is limited [22]. In this context, the present study aims to contribute to the current literature by investigating the potential link between the physical changes associated with acromegaly and the development of alexithymia, its associated psychopathological outcomes, and its impact on quality of life.

## Materials and methods

### Participants and study procedure

**Fig. 1:**
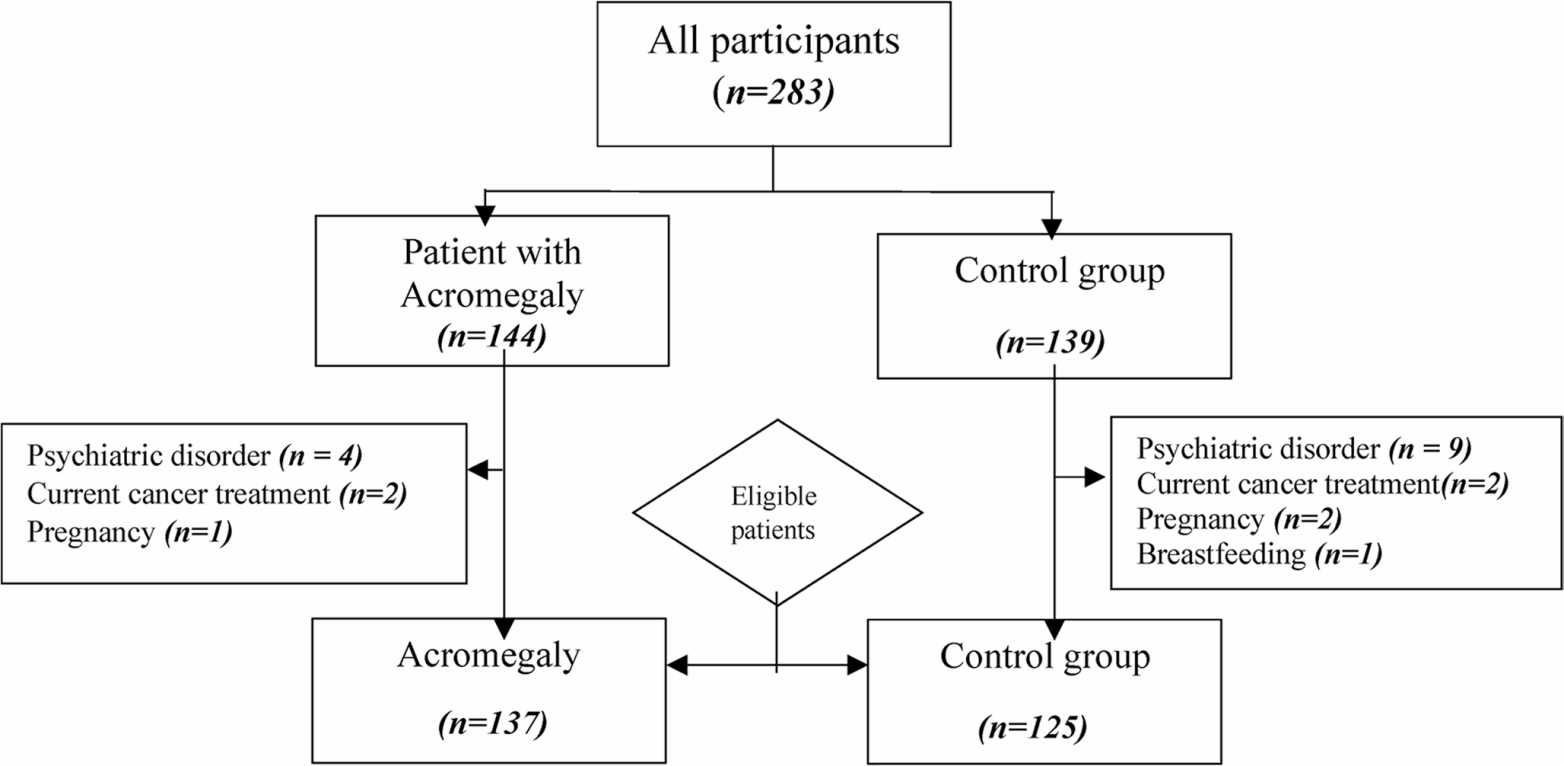
The flowchart showing the patients and controls’ recruitment process

A total of 144 consecutive patients with acromegaly who were followed up at the Division of Endocrinology-Metabolism, and Diabetes, Istanbul University-Cerrahpasa, Cerrahpasa Faculty of Medicine between 2009 2024 were included in the study. Exclusion criteria were: Patients with a diagnosis of psychiatric disease, patients currently receiving chemotherapy or radiotherapy for malignant disease, pregnant, and breastfeeding women (Fig. 1). In 137 patients with acromegaly who met the eligibility criteria, demographic data such as age, gender, marital status, degree, as well as time from symptom to diagnosis, disease duration, preoperative adenoma size, adenoma size at last visit, number of pituitary surgeries, presence of recurrence or residuals, history of radiotherapy, concomitant diabetes mellitus, hypertension, hyperlipidemia were collected from the medical records of the patients. In addition, acromegaly, hormone status and medication were noted. Enzyme-linked immunosorbent assay (ELISA) was used to determine IGF-1 and electrochemiluminescence was used for other hormone tests. Serum random GH, IGF-1, TSH, fT4, FSH, LH, total testosterone levels were measured in the morning after an overnight fast during that visit.

A questionnaire was administered to volunteers to assess the presence of any known chronic diseases. The patients currently receiving chemotherapy or radiotherapy for malignant disease, pregnant and breastfeeding women, and those diagnosed with a psychiatric disorder were excluded from participation. A total of 125 control participants were selected, matched based on age, sex, educational level, marital status, and the presence of chronic conditions, comorbidities such as diabetes, hypertension, hypothyroidism.

Remission status was classified as surgical (remission achieved with surgery alone) or medical (post-surgical remission maintained with medical therapy).

### Measures

#### Toronto Alexithymia Scale-20 (TAS-20)

TAS-20 is a 20-item scale that includes questions. Scores range from 1 to 5 for responses ranging from strongly agree to strongly disagree. In addition to the TAS-20 yields scores for three subscales: difficulty identifying feelings (DIF, total score for 7 items, potential range from 7 to 35), difficulty describing feelings (DDF, total score for 5 items, potential range 5–25), and externally oriented thinking (EOT, total score for 8 items, potential range 8–40). 61 was used as cut-off value in TAS-20 score. ≥61 was indicated as alexithymia [18, 23, 24]. The TAS-20 was used in the Turkish population and its validity and reliability were confirmed [25, 26].

#### Body Image Scale (BIS)

The scale consists of 40 items answered on a 5-point Likert scale. The higher the score, the higher the positive evaluation. A total score of 135 or less is considered to reflect impaired body image [27, 28]. 

#### Hospital Anxiety and Depression Inventory (HAD)

This scale consists of 14 questions and has been tested for validity and reliability in the Turkish population. The odd- numbered questions are calculated to assess anxiety and the even-numbered questions to assess depression. An anxiety score of 10 and above is considered generalized anxiety and a depression score of 7 and above is considered depression [29, 30].

#### Short Form-36 (SF-36)

The assessment is Likert-type except for some items. It consists of eight subscales, at the same time, Physical Component Summary Scale (PCS) was calculated as the total of physical subscores and Mental Component Summary Scale (MCS) was calculated as the total of social subscores. The subscales rate health on a scale of 0-100, with 0 indicating poor health and 100 indicating good health. The Turkish validity and reliability of the questionnaire consisting of 36 questions to assess quality of life was conducted by Kocyigit et al. [31, 32].

#### Acromegaly Quality of Life Scale (AcroQoL)

Every question is scored from 1 to 5. A score of 110 reflects the best possible quality of life. These scales have been *validated in Turkish populations* [33, 34].

### Statistical analysis

Statistical analyses were performed using the Statistical Package for the Social Sciences (SPSS) software (version 26.0). Data were first assessed for normality using the Kolmogorov–Smirnov test. Continuous variables were expressed as mean ± standard deviation (SD) for normally distributed data and median [interquartile range, IQR] for non-normally distributed data. Categorical variables were presented as frequencies and percentages. Comparisons between two independent groups were conducted using the independent samples t-test or the Mann–Whitney U test, depending on data distribution. Correlation coefficients between continuous variables were assessed using Spearman’s rank-order correlation and Pearson’s correlation test, as appropriate. Categorical variables were compared using Pearson’s chi-square test and Fisher’s exact test. A two-sided p-value < 0.05 was considered statistically significant, and all results were interpreted within a 95% confidence interval. Data visualization was performed using boxplots and bar graphs to illustrate group differences and distribution trends.

## Results

The sociodemographic characteristics of the participants are listed in Table 1. The disease duration of acromegaly patients was 94 [60–132] months. The clinical follow-up characteristics and disease-related parameters are also listed in Table 2.

**Table 1:** Sociodemographic and comorbidities characteristics of participants

	Acromegaly n=137)	Controls (n=125)	
	*n (%)*	*n (%)*	*P*
Age, *years Mean±SD*	47.68 *±* 11.09	45.16*±* 9.33	0.13
Sex			
Female	82 (59.9)	77 (61.6)	0.773
Male	55 (40.1)	48 (38.4)	
Marital status			
Married	109 (79.5)	95 (76)	0.593
Single	23 (16.7)	28 (22.4)	0.641
Divorced	5 (0.3)	2 (0.1)	0.65
Years of education			
<12 *years*	96 (70)	91 (66.4)	0.886
>12 *years*	41 (30)	34 (27.2)	0.902
Comorbidities			
Diabetes Mellitus	38 (27.7)	29 (23.2)	0.484
Hypertension	23 (16.7)	22 (17.6)	0.962
Hypothyroidism	21 (15.3)	13 (10.4)	0.317

**Table 2:** Clinical follow-up characteristics and disease-related parameters in patients with acromegaly

Patients (n)	137
Age at diagnosis *years* Mean ± SD	39.8 ± 10.88
Median [IQR]	
IGF-1 values at diagnosis	580.1 [400-882]
GH random values at diagnosis (mg/mL)	7.7 [4.14-19.8]
GH nadir post OGTT at diagnosis	4.4 [1.5-10.3]
IGF-1(ng/mL)	180 [135.2-226.7]
GH random value	1.4 [0.64-4.05]
TSH (μIU/mL)	1.71 [1.11-2.48]
fT4 (μIU/mL)	1.2 [1.05-1.32]
FSH (IU/L)	7.47 [3.99-13.4]
LH (IU/L) Total Testosterone (ng/dL)	5.86 [3.86-12.45] 376.8[289.2-546.5]
n (%)	
Adenoma size at diagnosis	
Microadenoma	27 (19,7)
Macroadenoma	88 (64.2)
Treatment	
None	46 (33.7)
Somatostatin analogues	74 (54)
Pegvisomant	10 (7)
Cabergoline	7 (5)
Radiotherapy	4 (2,9)
Remission Status	
Postoperative remission	41 (29.9)
Medical remission	91 (66.4)
Active disease	5 (3)

IGF1: Insulin Growth Factor-1, GH: Growth Hormone, OGTT: oral Glucose Tolerance test, IQR: interquartile range, Sd: standard deviation

**Fig. 2:**
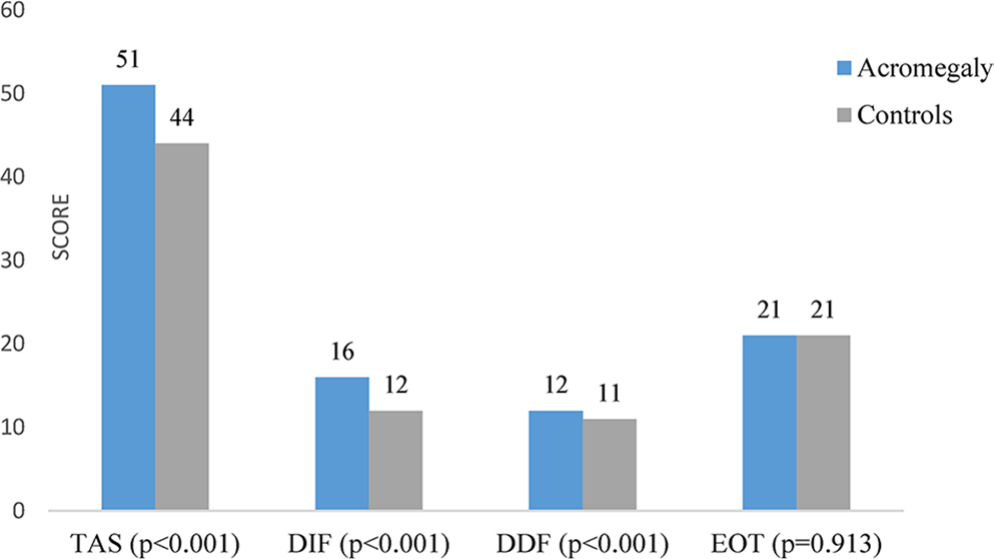
Comparison of Toronto Alexithymia Scale (TAS-20) total and subscale scores Difficulty Identifying Feelings, Difficulty Describing Feelings, and Externally Oriented Thinking between patients with acromegaly and healthy controls. TAS: Toronto Alexithymia scale, DIF: Difficulty Identifying Feelings, DDF: Difficulty Describing Feelings, EOT: Externally Oriented Thinking

In the acromegaly group, TAS-20 total scores, as well as the DIF and DDF subscale scores, were higher compared to the control group (51 vs. 44, *p* < 0.01; 16 vs. 12, *p* < 0.01; and 12 vs. 11, *p* < 0.01, respectively). DIF and DDF scores were markedly higher in patients with acromegaly compared to the control group, whereas EOT scores were similar between the two groups (21 vs. 21 *p* = 0.913) (Fig. 2). Alexithymia is present in 16.8% of patients with acromegaly, while it is present in 3.2% of individuals in the control group (*p* < 0.001). Although the prevalence of alexithymia was lower in patients with surgical remission than in those with medical remission, the difference did not reach statistical significance (9.8% vs. 16.5%, *p* = 0.278).

**Fig. 3:**
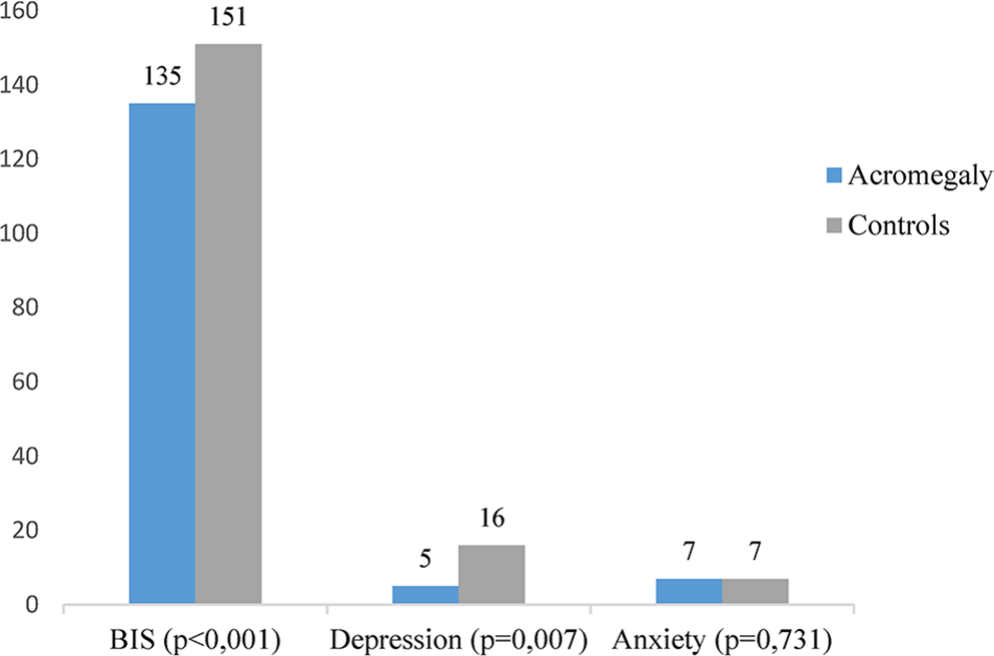
Comparison of Body Image Scale scores and Hospital Anxiety and Depression Scale subscale scores (depression and anxiety) between patients with acromegaly and healthy controls. BIS Body Image Scale

Patients with acromegaly had lower body image scores compared to control group (135 vs. 151, *p* < 0.05 Fig. 3). In addition, impaired body image was more frequently observed in the acromegaly group (43.7%) than in the control group (19.2%) (*p* < 0.01). Although Body Image Scale (BIS) scores were lower in women (134.75 ± 31.23) than in men (140.18 ± 26.96), the difference did not reach statistical significance (*p* = 0.357).

According to the Hospital Anxiety and Depression Scale (HADS), patients with acromegaly exhibited significantly higher levels of depressive symptoms compared to control group, whereas anxiety levels did not differ substantially between the two groups (Fig. 3). Depressive symptoms were present in 40% (*n* = 54) of patients with acromegaly and 25% (*n* = 32) of the control group (*p* = 0.025). In contrast, anxiety was reported in 29.1% (*n* = 40) of patients with acromegaly and 20% (*n* = 25) of controls, a difference that did not reach statistical significance (*p* = 0.114).

Patients with acromegaly exhibited reduced SF-36 scores relative to controls, apart from the physical role functioning and bodily pain subscales (Table 3). In patients with acromegaly, the general health perception subscale of the SF-36 differed between the surgical and medical remission groups (65 [45–80] vs. 50 [35–73], *p* = 0.049) No notable differences were identified in the remaining scale scores, including the TAS-20 subscales (DDF, DIF, EOT) (Table 4).

**Table 3:** Total and subscales scores of SF-36.

	Acromegaly	Controls	*P*
	Median [IQR		
SF-36 total	65.15 [44.62-81.58]	75.025 [59.25-84.07]	0.004
PCS	73.12 [48.75-87.5]	78.12 [62.18-87.81]	0.04
MCS	61.55 [42.82-76.05]	72.37 [54.7-81.58]	0.001
Phyicial Functioning	80 [60-95]	85 [70-95]	0.015
Physical Role Functioning	75 [25-100]	100 [50-100]	0.072
Bodily pain	77.5 [49.3-100]	77.5 [57.5-90]	0.366
General Health Perception	60 [40-75]	65 [55-80]	0.005
Vitality/Energy	50 [30-70]	60 [45-75]	0.001
Social functioning	75 [50-100]	87.5 [62.5-100]	0.037
Emotional Role Funtioning	66.7 [33.3-66.7]	66.7 [33.3-100]	0.005
Mental Health	68 [52-80]	76 [60-80]	0.005

PCS:Physical Component Summary Scale MCS:Mental Component Summary Scale IQR: Interquartile Range

**Table 4:** Alexithymia scores for patients with acromegaly in remission

	Remission with Surgical	Remission with Medical	
	*N=41*	*n=91*	
	Median [IQR]		*P*
TAS	50 [40-56]	58 [46,25-66,25]	0.108
DIF	15 [12-19]	17 [12-26]	0.08
DDF	12 [10-14]	14 [10-16,25]	0.362
EOT	21 [18-23]	25 [20-27]	0.286

TAS: Toronto Alexithymia scale, DIF: Difficulty Identifying Feelings, DDF: Difficulty Describing Feelings, EOT: Externally Oriented Thinking IQR: Interquartile Range

**Fig. 4:**
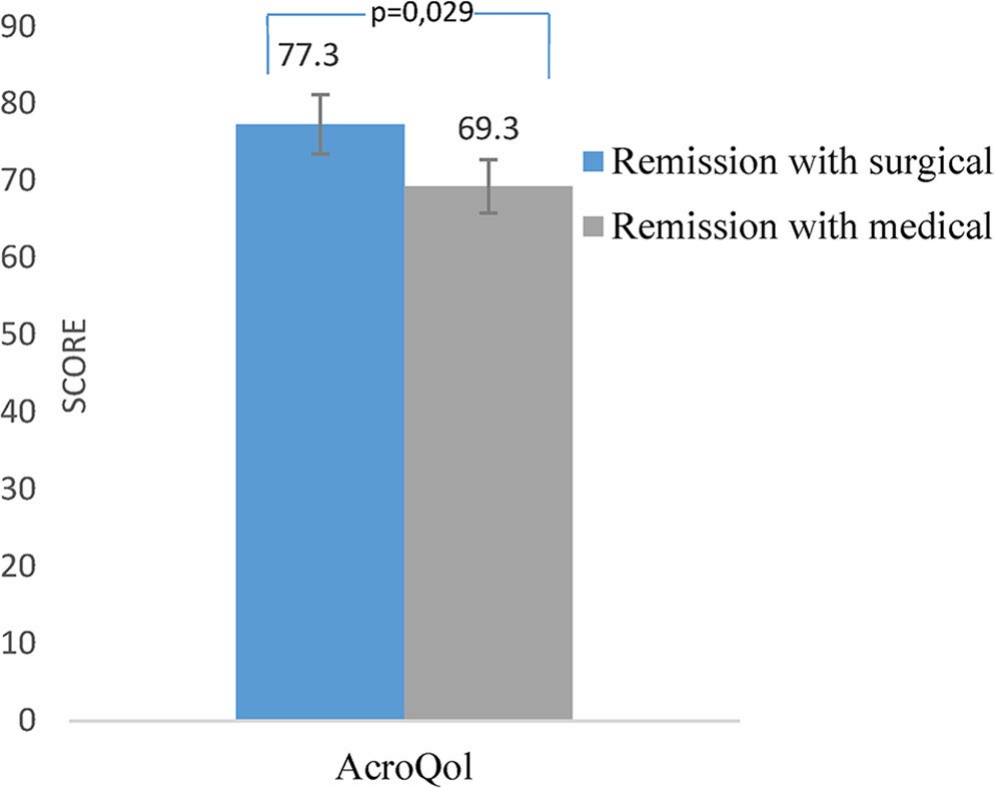
Evaluation of quality of life (AcroQoL) among patients in remission achieved through surgical or medical treatment. AcroQol: Acromegaly Quality of life Questionnaire

The Acromegaly Quality of Life Questionnaire (AcroQoL) scores were elevated in patients who had achieved surgical remission relative to those undergoing medical remission (Fig. 4). The type of somatostatin receptor ligand used did not appear to be associated with variation in quality of life outcomes (*p* = 0.56).

A comparison of GH levels between surgical and medical remission groups is presented in Table 5. Overall, GH levels were significantly lower in the surgical remission group at most evaluated timepoints.

**Table 5:** Comparison of GH Levels Between Surgical and Medical Remission Groups

Variable (ng/mL)	Remission with Surgical		Remission with Medical	p value*
Median [IQR]				
Preoperative GH		6.35 [3.21–10.32]	11.18 [4.91–30.95]	0.007
Preoperative OGTT nadir GH		3.24 [1.30–6.92]	6.68 [1.81–17.10]	0.076
Postoperative 3rd Month GH		0.50 [0.25–1.16]	3.64 [1.23–6.71]	<0.001
Postoperative 3rd Month OGTT nadir GH		0.15 [0.10–0.27]	1.39 [0.58–2.61]	<0.001
Current GH		0.77 [0.20–2.10]	1.91 [0.79–5.95]	<0.001

GH: Growth Hormone, OGTT: oral Glucose Tolerance test

Among male patients, 17 of 55 (30.9%) met the criteria for hypogonadism (total testosterone < 300 ng/dL). Subgroup analysis revealed no significant differences in alexithymia scores, body image measures, depressive and anxiety symptoms, or quality of life parameters between hypogonadal and eugonadal men (Table 6).

**Table 6:** Comparison of TAS-20 Scores According to Hypogonadism Status in Male Patients with Acromegaly

Parameter	Hypogonadism (*N=17)*	Eugonadis *(N=38)*	p-value
	Median [IQR]		
TAS-20 Total	50.0 [40.0–55.5]	49.0 [44.0–58.0]	0.437
DIF	16.0 [12.0–18.5]	17.0 [13.0–18.0]	0.634
DDF	12.0 [9.0–14.5]	14.0 [11.0–16.0]	0.269
EOT	20.0 [19.0–22.0]	21.0 [18.0–25.0]	0.346

TAS: Toronto Alexithymia scale, DIF: Difficulty Identifying Feelings, DDF: Difficulty Describing Feelings, EOT: Externally Oriented Thinking IQR: Interquartile Range

A positive correlation was found between alexithymia and both depression and anxiety scores (*r* = 0.469, *p* < 0.001; *r* = 0.377, *p* < 0.01, respectively). In contrast, alexithymia was negatively correlated with SF-36, AcroQoL and BIS (*r* = − 0.426, *p* < 0.01; *r* = − 0.465, *p* < 0.01; *r* = − 0.364, *p* < 0.01, respectively).

## Discussion

This study highlights a significantly higher prevalence of alexithymia in patients with acromegaly compared to control group. Remarkably, elevated alexithymia levels persisted even in patients who were in biochemical remission, suggesting that emotional dysregulation may be independent of active disease status. Additionally, patients with acromegaly exhibited impaired body image perception and increased depressive symptoms, despite no significant differences in anxiety levels. Overall, these findings point to a broader deterioration in both physical and mental health. Notably, patients who achieved remission through surgical treatment reported better quality of life outcomes than those managed with medical therapy alone.

The higher prevalence of alexithymia in patients with acromegaly suggests difficulty identifying and describing emotions, which may contribute to psychological distress and adverse mental health outcomes. Previous studies have demonstrated increased alexithymia in chronic illnesses, including diabetes, Hashimoto’s thyroiditis, and moderate to severe psoriasis [35–37]. However, unlike their findings, which reported no significant association between alexithymia and emotional symptoms such as anxiety or depression, our study found a significant positive correlation between alexithymia and these symptoms in patients with acromegaly. Similarly, a study by Sampogna et al. involving patients with moderate to severe psoriasis found that alexithymia levels decreased following clinical remission [37]. In contrast, our findings revealed persistently elevated alexithymia scores in patients with acromegaly, even after normalization of IGF-1 levels.

In untreated active acromegaly, mild-to-moderate cognitive impairment and neurophysiological alterations have been reported, possibly related to prolonged GH and IGF-1 excess [38, 39]. Nevertheless, whether these neurobiological changes directly translate into specific psychopathological conditions remains uncertain. Psychological symptoms may persist despite biochemical remission, and both GH excess and subsequent GH deficiency have been associated with impaired quality of life [6, 39–42], suggesting that dysregulation of the GH axis rather than absolute circulating GH levels alone may be relevant. In our cohort, the median disease duration was approximately 8–9 years, indicating long-standing exposure prior to evaluation. Although surgical remission was associated with lower GH levels than medical remission, no differences were observed in alexithymia or other psychological measures. As cumulative lifetime GH exposure was not quantified, long-term neurobiological effects cannot be excluded. Taken together, while a central neurobiological contribution of GH excess is biologically plausible, our findings do not support a simple linear relationship between circulating GH levels and psychopathological manifestations. The psychological burden in acromegaly appears to be multifactorial and not solely determined by current biochemical control.

In our study, we observed that individuals with acromegaly had significantly more impaired body perception compared to control group. Moreover, female patients reported worse body image than male patients. This may be explained by greater psychosocial sensitivity and societal pressures related to physical appearance among women. Consistent with our findings, Zhang et al. reported that among 68 patients with acromegaly, concerns regarding body image were common and were closely associated with a lower quality of life [13].

In a systematic review, Silvestro et al. reported depression rates ranging from 30% to 50% and anxiety rates from 20% to 40% in patients with acromegaly [22, 43]. In line with these findings, our study also demonstrated high levels of both depressive and anxiety symptoms, with depressive symptoms being more prevalent. The predominance of depression may be explained by several disease-specific factors, including the typical diagnostic delay of approximately 5–10 years, prolonged exposure to hormonal excess, the development of disease-related complications, chronic physical changes with subsequent body image disturbance, and the long-standing course of the disease, all of which may predispose patients to more sustained depressive symptomatology. In contrast, anxiety symptoms are often more closely related to acute or situational stressors, which may account for their relatively lower prominence in chronic endocrine conditions. Furthermore, Uysal and colleagues showed that depressive symptoms in patients with acromegaly adversely affect their cohabitants, leading to a decline quality of life [44]. This underscores how the emotional burden of acromegaly may extend beyond the individual to impact close social relationships.The higher prevalence of alexithymia observed in our acromegaly cohort suggests that difficulties in identifying and expressing emotions may not only contribute to psychological distress, but also impair interpersonal communication.

In the present study, patients with acromegaly reported poorer quality of life according to the SF-36 questionnaire. Similarly, Oliveira et al. assessed the quality of life in patients with acromegaly using both a general (SF-36) and a disease-specific (AcroQoL) questionnaire, and found lower scores across all subscales except social functioning. Using the same instruments, we also observed significantly impaired quality of life in our cohort, particularly in domains other than physical role functioning and bodily pain [45].

Another study evaluating the quality of life before and after surgery using the SF-36 demonstrated that surgical intervention led to improvements in quality of life [46]. However, persistent limitations were noted in emotional role functioning and mental health domains, suggesting that psychological support may still be required even after remission is achieved. In our study, overall quality of life remained poor, except for bodily pain and physical functioning. Furthermore, when evaluated using the AcroQoL scale, patients in remission after surgery reported better quality of life than those in remission with medical therapy.These findings suggest that achieving remission through surgery may contribute more positively to overall life satisfaction than medical treatment alone. Taken together, these results highlight the importance of addressing quality of life alongside biochemical disease control in the comprehensive management of acromegaly.

In the literature, only a limited number of studies have specifically examined alexithymia, anxiety, and depressive symptoms in patients with acromegaly and their impact on quality of life. In other male hypogonadal populations, depressive symptoms, anxiety, and impaired quality of life have been frequently reporte [47–50]. However, data directly linking hypogonadism to alexithymia remain inconsistent. In our cohort, no significant differences were observed between hypogonadal and eugonadal male patients with acromegaly regarding alexithymia, mood symptoms, or quality of life measures. Taken together, these findings suggest that gonadal status alone may not independently account for emotional processing difficulties in acromegaly.

This study has several limitations. First, as is common in survey-based research, the reliance on self-reported data may not accurately reflect the participants’ actual clinical status. Second, the retrospective design of the study limits the ability to establish causal relationships or assess changes over time. Third, female gonadal status could not be reliably evaluated, as estradiol levels were measured at random time points rather than standardized to the follicular phase of the menstrual cycle; therefore, the association between female hypogonadism and psychological outcomes could not be examined. Finally, the number of patients with active, non-remission disease was small (*n* = 5), which limited meaningful comparisons between controlled and uncontrolled acromegaly.

The present study demonstrates that alexithymia is more prevalent in patients with acromegaly compared to healthy individuals and often persists even after remission. This finding supports the notion that alexithymia may serve as a predictive marker for adverse psychiatric outcomes such as depression and anxiety. Our results highlight the significant psychological burden of chronic illness and its negative impact on patients’ quality of life. These findings underscore the importance of a comprehensive treatment approach to acromegaly one that addresses not only hormonal regulation but also incorporates thorough psychological assessment and ongoing psychosocial support.

## Data Availability

Some or all of the datasets generated and analysed during the current study are not publicly available, but are available from the corresponding author upon reasonable request.
